# Major histocompatibility complex peptide ligands as olfactory cues in human body odour assessment

**DOI:** 10.1098/rspb.2012.2889

**Published:** 2013-03-22

**Authors:** Manfred Milinski, Ilona Croy, Thomas Hummel, Thomas Boehm

**Affiliations:** 1 Department of Evolutionary Ecology, Max Planck Institute of Evolutionary Biology, August-Thienemann-Strasse 2, 24306 Ploen, Germany; 2 Department of Otorhinolaryngology, University of Dresden Medical School, Fetscherstrasse 74, 01307 Dresden, Germany; 3 Department of Occupational and Environmental Medicine, University of Gothenburg, Medicinaragatan 16, 40530 Gothenburg, Sweden; 4 Department of Developmental Immunology, Max Planck Institute of Immunobiology and Epigenetics, Stuebeweg 51, 79108 Freiburg, Germany

**Keywords:** mate choice, major histocompatibility complex, fMRI

## Abstract

In many animal species, social communication and mate choice are influenced by cues encoded by the major histocompatibility complex (MHC). The mechanism by which the MHC influences sexual selection is a matter of intense debate. In mice, peptide ligands of MHC molecules activate subsets of vomeronasal and olfactory sensory neurons and influence social memory formation; in sticklebacks, such peptides predictably modify the outcome of mate choice. Here, we examine whether this evolutionarily conserved mechanism of interindividual communication extends to humans. In psychometric tests, volunteers recognized the supplementation of their body odour by MHC peptides and preferred ‘self’ to ‘non-self’ ligands when asked to decide whether the modified odour smelled ‘like themselves’ or ‘like their favourite perfume’. Functional magnetic resonance imaging indicated that ‘self’-peptides specifically activated a region in the right middle frontal cortex. Our results suggest that despite the absence of a vomeronasal organ, humans have the ability to detect and evaluate MHC peptides in body odour. This may provide a basis for the sensory evaluation of potential partners during human mate choice.

## 1. Introduction

Major histocompatibility complex (MHC) molecules are involved in antigen presentation and their structure determines the probability with which a given pathogen will be recognized by the individual's immune system [1]. Because MHC molecules critically influence the susceptibility to infection, maintenance of a sufficient degree of MHC diversity in natural populations is a key survival parameter in the face of constantly changing pathogen spectra [2–5]. Behavioural mechanisms that guide non-random mating based on MHC genotypes are considered to be a means by which an optimum degree of individual MHC diversity is maintained in the offspring. While there is strong experimental support for MHC-associated behaviour in animals [5–9], including non-human primates [10,11], the situation in humans is more complex. Evidence in favour of MHC-associated behaviour has emerged from studies on the sexual interest of females [12] and their preferences for certain male body odour [13,14]; studies on the degree of genetic relatedness of mated and unmated couples of the opposite sex have produced mixed results, suggesting a role for MHC genotype in some [15,16] but not all populations [17].

In a double-blind study, Wedekind *et al.* [14] found that women preferred the odour of shirts worn by men with different MHC alleles to those of men with more closely matching MHC alleles; a similar observation was made with male participants [18]. These findings (reviewed in [12]) suggested a relationship between MHC-dependent odour signalling and preference for a specific personal perfume. In a subsequent study with the MHC-typed student cohort originally tested by Wedekind *et al.* [14], Milinski & Wedekind [19] tested this prediction directly; participants who shared MHC alleles expressed a strong preference for the same natural perfume ingredient for use on themselves, but not on a potential partner. Thus, because MHC genotypes determine individual perfume preferences, it appears that perfumes function as amplifiers of MHC-related individual body odours [20]. Indeed, a recent study showed that self-preferred perfumes added to body odour are preferred to perfumes allocated by the investigator and added to the same body odour [21]. Collectively, these findings may provide an explanation for the fact that humans of all cultures have used fragrances for at least 5000 years and for the observation that persistent interindividual differences exist for the preference of certain perfumes [19].

Recent work on the molecular nature of the chemosensory stimuli underlying MHC-associated behaviour in animals indicates that peptide antigens presented by MHC molecules act as olfactory cues in different species in addition to their well-known function in eliciting immune responses [22–25]. Here, we have examined the ability of humans to recognize and evaluate the modification of their body odour by allele-specific MHC peptide ligands. The present psychophysical and neurophysiological studies suggest that MHC peptide ligands convey information about the MHC genotype and may thus represent at least part of natural MHC-dependent human body odour signals.

## 2. Material and methods

### (a) Study participants

Female students were recruited from the Universities of Hamburg and Kiel. They were genotyped for human leucocyte antigen (HLA) HLA-A and HLA-B [26] at the University Hospital Hamburg using the reverse SSO line blot assay (Dynal Reli SSO, Invitrogen); note that for historical reasons, the products of the human MHC locus are designated as HLA. This analysis revealed that 19 were positive for HLA-A*02, six were positive for HLA-A*24 (of which two were also positive for HLA-A*02) and nine carried neither HLA-A*02 nor HLA-A*24. In one participant (no. 6), the presence of the A*3002 allele was considered to be functionally equivalent to A*2402, because their peptide specificity is identical [27]. Sixteen participants carried one or more alleles (in addition to HLA-A*02 or HLA-A*24) with unknown peptide specificity, which were considered to be ‘non-self’; however, given the diversity of residues at anchor positions, the probability that such a ‘non-self’-peptide has the quality of ‘self’ is much less than 0.5; in this case, the effect on our results would be conservative, that is, it would weaken any observed effect. Four participants carried alleles whose peptide specificities are not precisely known, but are unlikely to be identical to those used as a stimulus peptide; if not, their influence would diminish rather than increase the observed effects, because scores would then be given the wrong sign. At the time of performing the psychometric tests, the age of participants was 25.9±0.9 years (mean±s.e.; *n* = 25; range 21–36 years). Details of the study population are summarized in the electronic supplementary material, table S1.

### (b) Design of peptide ligands

On the basis of known ligand specificities [27], nonamer peptides were synthesized and purified by Thermo Fisher Scientific GmbH (Ulm, Germany). The peptides and their cognate HLA alleles are listed in the electronic supplementary material, table S2.

### (c) Psychometric test procedure

Participants were provided with a perfume-free body soap, an untreated cotton T-shirt and two pairs of two bottles per test day containing synthesized HLA ligand peptides, specific for either HLA A*0201 or HLA A*2402 (0.005 mM in phosphate-buffered saline (PBS)), or solvent (PBS). After a shower using the body soap and wearing the T-shirt overnight, the participant put four drops from bottle 1 in her left hand and rubbed them under her right armpit, then put four drops from bottle 2 in her right hand and rubbed them under her left armpit. In mice [22] and sticklebacks [28], MHC peptide ligands elicit a behavioural response only when accompanied by a natural validating factor. We assumed that validation is also necessary in humans and that it is likely to be produced by certain glands in the armpit [29–31]. The participant was then asked to evaluate the smell of each of her armpits by sniffing repeatedly from a close distance and to decide whether one side, and if so, which side, smells like herself or a perfume she would like to smell on herself (‘would you like to smell like this?’). She marked on a questionnaire ‘I prefer for myself the smell of my left armpit/my right armpit; I do not smell a difference; if I have a preference, it is weak, medium or strong.’ The following night, wearing the same T-shirt, the protocol was repeated with bottles 3 and 4; this time sides allocated to peptide and solvent were swapped in order to control for possible side effects [32]. In the questionnaire, the participants were asked about their smoking habits, their use of contraceptive medication, and whether they had a cold during the test. Participants took part in two to six of such test sessions depending on availability at intervals of at least three months.

### (d) Data analysis for psychometric test

The psychometric tests were carried out in a double-blind fashion and the results of the genotype were revealed only after completion of the tests. The scores assigned to ‘non-self‘ stimuli were subtracted from scores given to ‘self’ stimuli (resulting in positive values when self is preferred to non-self (see text for details)); the following scores could be given: no preference, 0; weak preference, 1; medium preference, 2, strong preference, 3. Data from each participant were averaged and only the average entered into the analysis to avoid pseudo-replication.

### (e) Functional magnetic resonance imaging study: participants

Twenty-two right-handed women participated in the functional magnetic resonance imaging (fMRI) study; medical histories indicated that all participants were in good health. Olfactory function, assessed by means of ‘Sniffin Sticks’, (Burghart, Wedel, Germany) [33] was compatible with a normal sense of smell for all but one person who was excluded from the analysis; two further participants had to be excluded from the analysis, one because of pronounced brain abnormalities, the other because of technical problems with the dataset. Data from participants 15–33 (see the electronic supplementary material, table S1 for details of the study population) were processed. At the time of the fMRI studies, the age of participants was 27±1.1 years (mean±s.e.; *n* = 19; range 18–35 years).

### (f) Functional magnetic resonance imaging procedure

The study was performed with a 1.5 T MR-scanner (Sonata; Siemens, Erlangen, Germany). Peptide solutions were prepared from lyophilized stocks at a final concentration of 25 mM in PBS. Peptide and mock control solutions were prepared using the same type of plastic ware (50 ml polypropylene Falcon tubes; BectonDickinson). Two peptide solutions, the solvent control, and an additional odour (peach) were presented to both nostrils in a total of eight sessions. Peach is an odour known to cause reliable activation in olfactory-relevant areas and was therefore used as a control to ascertain the validity of the experiment; the solvent without peptides also generated activation of olfactorily relevant areas, presumably owing to contaminants in the plastic containers used. The order of the sessions using peptides or solvent was counterbalanced between participants. Peach was always presented in sessions 7 and 8. To focus their attention on self-assessment, participants were instructed that, after each session, they would be asked to rate the quality of odours on a scale of 0–10 (0, I would not like to smell like this at all; 10, I would very much like to smell like this). Each experimental session comprised six on/off blocks lasting 20 s each. Participants were blind to compound identity. The odours were applied to the participants using a computer-controlled olfactometer (Sommer, Mannheim; Germany). Stimuli were embedded in a constant flow of odourless air (total flow 2 l min^−1^). The stimuli were directed through a small tube from the olfactometer to the participants' noses. During the ‘on’-blocks odourized air was intermittently (1 s air, followed by 2 s pause) delivered to the nasal cavity, at a rate of 2 l min^−1^. During the ‘off’-block, participants received pulses of odourless air. With respect to the intensity of the odours, participants reported no significant differences between solvent (8.4±3.6 (mean±s.d.)), self-peptide (9.4±4.7) and non-self-peptide (8.5±4.1) (scores, from undetectable odour (0) to very strong odour (20) (the scores from applications to both nostrils were totalled for the analysis); control peach odour intensity rating, 16.4±3.6). These results indicate that self-peptide and non-self-peptide stimuli were presented supraliminally. With respect to the preference as self-odours, no significant differences were observed when the participants gave their scores after the short exposure to each odour during the individual sessions of the fMRI experiment (solvent, 9.1±3.7; self-peptide, 7.3±3.5; non-self-peptide, 8.8±2.7). Note the different experimental design of the psychometric test that involves a comparative rather than an independent assessment of odours without time restrictions.

For functional brain activation data, 96 volumes per session were acquired by means of a 26 axial-slice matrix 2D spin-echo/echo-planar sequence (repetition time (TR): 2500 ms/echo time (TE): 40 ms, matrix = 64 × 64, voxel size 3 × 3 × 3 mm³). Following the fMRI sessions, a T1-weighted image was acquired by using a T1-MPR sequence (TR: 2180ms/TE: 3.9 ms; TI 1100 ms, matrix 352 × 384).

### (g) Functional magnetic resonance imaging data analysis

Data analysis was performed with SPM 8 software (Statistical Parametric Mapping; Wellcome Department of Imaging Neuroscience, Institute of Neurology at University College London, UK), implemented in Matlab R2007b (Math Works Inc., Natick, MA, USA), following spatial pre-processing with the same software (spatial filtering: high-pass filter 128 Hz, registering, realignment, co-registration between functional and structural images, normalization using segmentation procedure, smoothing by means of 6 × 6 × 6 mm^3^ FWHM Gaussian kernel). Motion parameters were included as covariates. Activation coordinates are presented in MNI space. SPM-matrices reflecting the ON–OFF differences were calculated for each session and participant. Analysis was based on the general linear modelling approach. Individual SPM-contrasts were subjected to a full-factorial second level analysis with the two conditions ‘side’ (two: left, right) and ‘substance’ (four: two peptides in solvent, solvent, additional odour (peach)). Whole brain analysis and small volume region of interest (ROI) analysis were performed for seven cortical areas previously reported to be related to self-processing: right middle frontal cortex, superior and inferior parietal cortex and fusiform cortex [34], right inferior frontal and anterior cingulated cortex and left insular cortex [35]. Masks were created using the aal atlas [36] embedded in the WFU PickAtlas v. 2.4 software [37]. Four *t*-contrasts were calculated (‘self’-peptide minus solvent, ‘self’-peptide minus ‘non-self’-peptide, ‘non-self’-peptide minus solvent, ‘non-self’-peptide minus ‘self’-peptide). A comparative analysis of activated brain areas resulting from exposure to two different olfactory stimuli tends to cancel out olfactory areas and instead highlights differentially activated regions only, such as those activated by either self or non-self-peptides. Note that the solvent control also activated olfactory regions, presumably owing to the presence of trace contaminants in the disposable plastic containers used throughout the study; therefore, subtractive analysis was considered the most reliable approach to reveal peptide-induced activation. Moreover, activation of a certain brain area was considered to be present only when signals were detected in a particular region for both self-peptide versus solvent and self-peptide versus non-self-peptide, or non-self-peptide versus solvent and non-self-peptide versus self-peptide, respectively. Analysis was based on *t*-tests with global height threshold *p* < 0.001, Bonferroni-corrected for the seven search areas and extent threshold of *k* = 3. Additionally, the family-wise error (FWE) rates for activations found within the search areas are presented.

## 3. Results

### (a) Psychometric assessment of body odour

In a first set of experiments with human volunteers, we examined self-assessment of natural body odour emanating from the armpits after supplementation with prototypic MHC peptide ligands. Hence, in contrast to previous studies, our experimental paradigm specifically focused on self-preference. As expected from HLA allele frequencies in the catchment area for our study population, 18 out of 22 participants were positive for either HLA-A2 or HLA-A24 alleles (or both; electronic supplementary material, table S1), justifying the use of two prototypic peptide ligands (SLLPAIVEL for HLA-A2 and KYPENFFLL for HLA-A24; electronic supplementary material, table S2) as self and non-self stimuli in the double-blind study design (see §2 for details). In individual test sessions, comparisons were made for peptide versus solvent, or A2 versus A24 peptides. The participants were asked to apply two different solutions to their left and right armpits on two consecutive days and then to compare the smell of both armpits (see §2 for details). In the solutions provided to the participants for the second day, the contents were exchanged relative to the previous session to control for potential side bias. Participants had to decide which armpit smelled ‘like themselves’ (or ‘like their favourite perfume’). They were also asked if their preference was weak, medium or strong, or whether they detected no difference between the two armpits.

**Figure 1.**
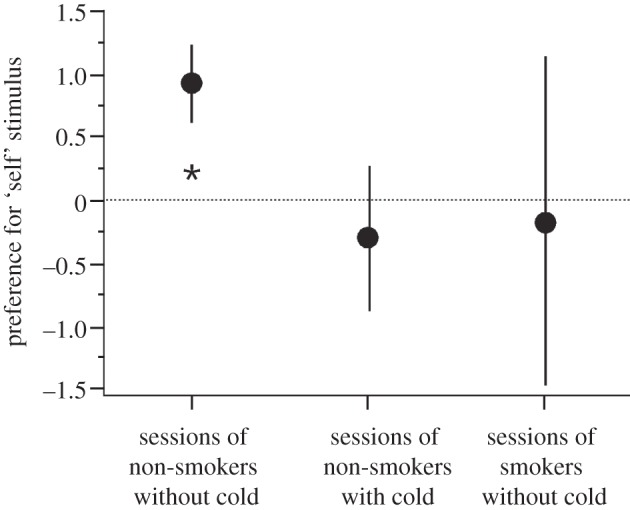
Preference for body odour supplemented with ‘self’ stimuli. Participants indicated preference on a scale from +3 to −3. Although participants took part in several trials, only the mean values were used to avoid pseudo-replication. Preference is shown for all sessions of non-smokers without a cold (left), non-smokers with a cold (middle), and smokers without a cold (right). Mean±s.e.m.; *p = 0.0167, two-tailed.

In the participants positive for HLA-A2, but negative for HLA-A24, the scores for the armpit exposed to HLA-A2 peptide (‘self’) were given positive values, the scores for the armpit exposed to HLA-A24 peptide or solvent (both ‘non-self’ relative to the HLA-A2 peptide) were given negative values. For the participants negative for HLA-A2, but positive for HLA-A24, only the scores for the armpits exposed to HLA-A24 peptide (‘self’) were given positive values. For HLA-A2/HLA-A24 double-positive participants, the scores for either peptide (‘self’) were given positive, those for solvent (‘non-self’) negative values; for HLA-A2/HLA-A24 double-negative participants, the scores for solvent were given positive (‘self’, relative to peptides) and the scores for either peptide (‘non-self’) negative values. We also recorded whether participants were smokers or had a cold when they carried out the test. Individual scores were averaged across all test sessions. When the data for all sessions of non-smokers without a cold were analysed (two-tailed Wilcoxon one-sample test; average preference compared with 0), a significant preference for the ‘self’ side was found (*n* = 17 participants (total number of trials = 37; average number of trials per participant = 2.2±0.3 s.e.m), *z* = −2.394, *p* = 0.0167, two-tailed; figure 1). When the sessions of the same cohort with a cold were examined, no significant difference was found (*n* = 12, *z* = −0462, *p* = 0.647, two-tailed); similarly, sessions of smokers without a cold failed to show a difference (*n* = 4, *z* = −0.365, *p* = 0.715, two-tailed; figure 1). Thus, the preference for ‘self’ (either self-peptide or solvent) to ‘non-self’ (either non-self-peptide or solvent) was clearly evident when all participants with potential impairment of their sense of smell (smoking and/or cold) [38,39] were omitted.

### (b) Functional magnetic resonance imaging

**Figure 2.**
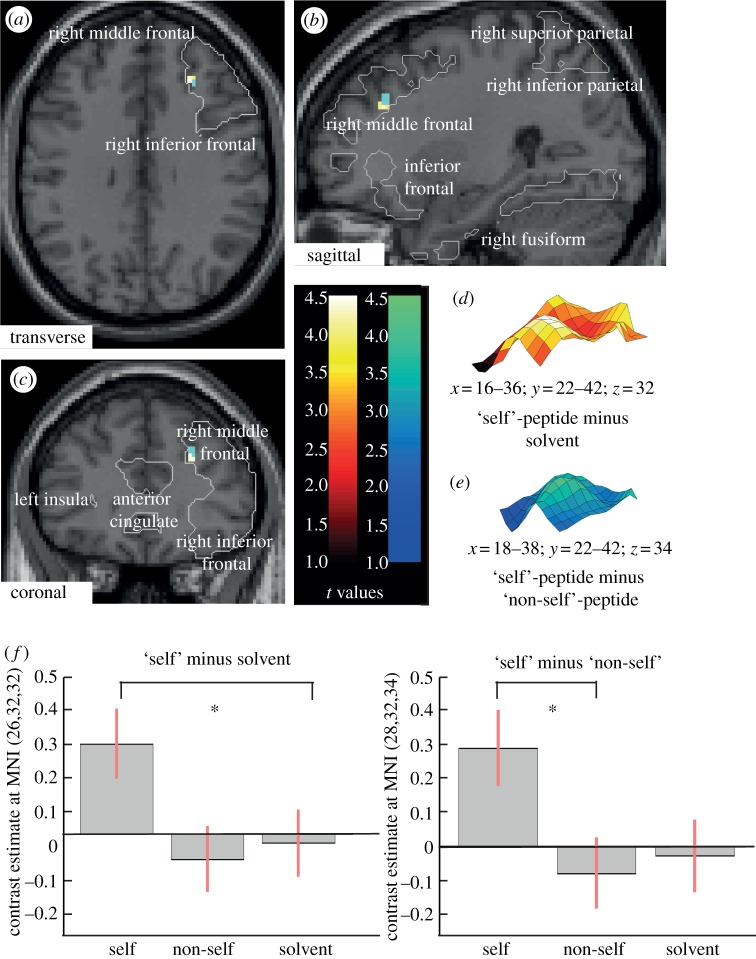
Activation of the right middle frontal cortex by ‘self’-peptides. Activated areas are visualized in a T1-weighted structural template. (*a*) Transverse section; this section encompasses parts of the right middle and inferior frontal cortex as ROI (outlined). The *t*-values for activations induced by ‘self’-peptides relative to solvent are indicated on a blue-to-green scale, those for ‘self’-peptides relative to ‘non-self’-peptides on a red-to-yellow scale. Note the co-localization of the activated regions. (*b*) Sagittal section with ROIs indicated. (*c*) Coronal section with ROIs indicated. (*d*) Spatial activation profile for ‘self’-peptide relative to solvent. The MNI coordinates are indicated as are the colour-coded *t*-values (*P*(FWE)corr = 0.074; height threshold was set to *p* < 0.001 (Bonferroni-corrected) and extend threshold to *k* = 3 (see (*f*) for contrast estimates). (*e*) Spatial activation profile for ‘self’-peptide relative to ‘non-self’-peptide. The MNI coordinates are indicated as are the colour-coded *t*-values (*P*(FWE)corr = 0.111); height threshold was set to *p* < 0.001 (Bonferroni-corrected) and extend threshold to *k* = 3 (see (*f*) for contrast estimates). (*f*) Contrast estimates for selected regions after stimulation with ‘self’-peptides. *,*p* < 0.001, Bonferroni-corrected. Activated areas are visualized in a T1-weighted structural template.

The results of the above psychometric tests indicate that human participants are capable of recognizing modifications of their body odour by MHC peptides. Our aim was to confirm this distinct perceptual capacity using fMRI. For this, peptides were delivered to the nostrils of study participants in aerosolized form, and the activation of particular brain areas was determined. To focus the attention of participants on self-assessment, they were asked to rate whether they preferred to smell like the presented odour and to repeat this after each session (see §2 for details). Eleven of 19 participants (all right-handed) included in this analysis had also participated in the psychometric tests (see the electronic supplementary material, table S1). Four different stimuli (solvent; ‘self’-peptide; ‘non-self’-peptide; peach odour as control) were delivered to both nostrils in eight consecutive sessions (see §2 for details). Peptide stimuli (see the electronic supplementary material, table S2) were selected according to the HLA genotype of the test participants (see the electronic supplementary material, table S1). ROI analysis was performed for seven cortical areas previously reported to be related to self-processing: right middle frontal cortex, superior and inferior parietal cortex and fusiform cortex, right inferior frontal and anterior cingulated cortex, and left insular cortex [34,35,40]. ‘Self’-peptides induced specific activation in the right middle frontal region, when compared with both solvent (Montreal Neurological Institute, MNI, coordinates (*x*,*y*,*z*) = 26,32,32; *P*_corr_ < 0.001) and ‘non-self’-peptides (MNI (*x*,*y*,*z*) = 28,32,34; *P*_corr_ < 0.001; figure 2). Middle frontal structures are known to be involved in cognitive self-representation [41]. By contrast, activation induced by ‘non-self’-peptides was much weaker, as expected from the ‘self’-centric paradigm of the study; in this case, *t*-values were lower and the overlap of activated regions resulting from comparisons of non-self-peptide versus solvent and of non-self-peptide versus self-peptide was less obvious (see the electronic supplementary material, figure S1). The peach odour elicited robust activation of olfactory brain areas (see the electronic supplementary material, table S3). Collectively, these results indicate that ‘self’-peptides elicit a response in a distinct brain region.

## 4. Discussion

The non-classical function of MHC peptides as activating cues for sensory neurons of the olfactory system provides a mechanistic explanation for the role of MHC alleles in guiding behavioural decisions in a variety of contexts and animal species. Our study suggests that MHC peptide ligands may play a similar role in humans. In a behavioural paradigm of self-preference, participants considered the modification of their body odour by ‘self’-peptides more desirable than the modification by ‘non-self’-peptides, indicating that MHC peptide ligands comprise a functionally relevant component of human body odour. These findings are in keeping with previous observations that humans sharing specific MHC alleles also share a preference for particular natural perfume ingredients [19] and posit that perfumes may contain structurally diverse peptide mimics. Interestingly, customers usually buy perfumes for their own use [42] and have always done so [43]. If perfumes are indeed chosen to reveal and/or enhance one's own body odour [20,21], it is not surprising that one dislikes on others what one likes for oneself [19]. This switch of choice preference with respect to perfume usage might be explained by ‘phenotype-matching’ [44], a process that is also implicated in kin-recognition.

Remarkably, exposure to MHC peptide ligands activated specific brain regions, indicating that humans, despite lacking a functional vomeronasal organ [45], possess the sensory facility to recognize the presence of MHC-associated olfactory cues. It is possible therefore that peptides activate sensory neurons located in the main olfactory epithelium, as was observed in mice [25]. Our results are compatible with the notion that the right middle frontal region is a multimodal convergence zone [40] that might provide the anatomical basis for self-referentiality by integrating various extero- and interoceptive inputs, including peptide stimuli. Notably, the activation of particular brain regions by exposure to peptides does not reflect the precise chemical structure of MHC peptides but rather their ‘self’ or ‘non-self’ qualities relative to the individual's MHC genotype. This suggests the presence of an internal reference for MHC genotype and is reminiscent of an equivalent facility in MHC-associated behavioural decisions in mice [22,25] and sticklebacks [24]. Hence, our study suggests that, as in mice and fish, sensory evaluation of MHC diversity through the recognition of structurally diverse MHC ligands may be involved in human MHC-associated behaviour.
